# Regular Exercise Modulates the *dfoxo*/*dsrebp* Pathway to Alleviate High-Fat-Diet-Induced Obesity and Cardiac Dysfunction in Drosophila

**DOI:** 10.3390/ijms242115562

**Published:** 2023-10-25

**Authors:** Hanhui Yan, Meng Ding, Tianhang Peng, Ping Zhang, Rui Tian, Lan Zheng

**Affiliations:** Key Laboratory of Physical Fitness and Exercise Rehabilitation of Hunan Province, College of Physical Education, Hunan Normal University, Changsha 410012, China; 201730171053@hunnu.edu.cn (H.Y.); 201820151262@hunnu.edu.cn (M.D.); 202120152827@hunnu.edu.cn (T.P.); 202220152989@hunnu.edu.cn (P.Z.); 202220152991@hunnu.edu.cn (R.T.)

**Keywords:** regular exercise, *dfoxo*/*dsrebp*, high-fat diet, obesity, cardiac dysfunction

## Abstract

Obesity is a prevalent metabolic disorder associated with various diseases, including cardiovascular conditions. While exercise is recognized as an effective approach for preventing and treating obesity, its underlying molecular mechanisms remain unclear. This study aimed to explore the impact of regular exercise on high-fat-diet-induced obesity and cardiac dysfunction in Drosophila, shedding light on its molecular mechanisms by identifying its regulation of the *dfoxo* and *dsrebp* signaling pathways. Our findings demonstrated that a high-fat diet leads to weight gain, fat accumulation, reduced climbing performance, and elevated triglyceride levels in Drosophila. Additionally, cardiac microfilaments in these flies exhibited irregularities, breakages, and shortening. M-mode analysis revealed that high-fat-diet-fed Drosophila displayed increased heart rates, shortened cardiac cycles, decreased systolic intervals, heightened arrhythmia indices, reduced diastolic diameters, and diminished fractional shortening. Remarkably, regular exercise effectively ameliorated these adverse outcomes. Further analysis showed that regular exercise reduced fat synthesis, promoted lipolysis, and mitigated high-fat-diet-induced cardiac dysfunction in Drosophila. These results suggest that regular exercise may mitigate high-fat-diet-induced obesity and cardiac dysfunction in Drosophila by regulating the *dfoxo* and *dsrebp* signaling pathways, offering valuable insights into the mechanisms underlying the beneficial effects of exercise on obesity and cardiac dysfunction induced by a high-fat diet.

## 1. Introduction

Obesity is a chronic metabolic disease, caused by a variety of factors, and is a significant global public health concern [1]. It is intricately linked to various chronic illnesses, including type 2 diabetes, cardiovascular disease, and fatty-liver disease. The development of obesity involves multiple factors, such as genetics, environment, and lifestyle, with dietary composition standing out as a crucial contributing factor [2]. High-fat diet (HFD) consumption can lead to an excessive energy intake, resulting in the proliferation and expansion of adipose tissues, as well as metabolic disruptions and chronic inflammatory responses within the organism [3].

Regular exercise represents one of the most effective strategies for both preventing and treating obesity and its associated complications [4]. It serves to elevate energy expenditure, diminish fat accumulation, rebalance various metabolic and signaling pathways in the body, mitigate chronic inflammatory responses, and safeguard cardiovascular function [5]. Nevertheless, the impact of regular exercise on high-fat-diet-induced obesity and cardiac dysfunction in Drosophila, along with its underlying molecular mechanisms, remains unexplored.

Drosophila, as a widely embraced model organism, possesses several advantages, including a conserved genome, ease of genetic manipulation, a short life cycle, and cost-effectiveness, rendering it invaluable for research on metabolic disorders [6]. Drosophila shares a high degree of homology with the *foxo* and *srebp* genes in mammals, which play pivotal roles in regulating cellular metabolism, responding to stress, and influencing aging processes [7,8].

Forkhead box O (*foxo*) proteins are responsive to insulin signaling and play key roles in regulating the oxidation and synthesis of sugars and fats [9]. Sterol regulatory element-binding protein (*srebp*), on the other hand, responds to sterol levels and governs the synthesis and uptake of cholesterol and fatty acids [10]. In mammals, complex interplay and regulatory relationships exist between *foxo* and *srebp*. On the one hand, foxo can inhibit the activation and function of *srebp* [11]. Conversely, *srebp* can also influence the expression and function of *foxo* [11]. *foxo* and *srebp* serve contrasting roles in lipid metabolism, with foxo typically regarded as an anti-adipogenic factor and *srebp* as a pro-adipogenic factor [12,13]. Consequently, *foxo* and *srebp* represent two crucial classes of transcription factors with divergent, and sometimes opposing, roles in lipid metabolism [14]. Investigating the molecular interactions between *dfoxo* and *dsrebp* can offer insights into the etiology and progression of diseases associated with adipose-metabolism disorders.

The primary objective of this study was to examine the impact of regular exercise on high-fat-diet-induced obesity and cardiac dysfunction in Drosophila, with a focus on unraveling the underlying molecular mechanisms mediated by the dfoxo and dsrebp signaling pathways. 

## 2. Results

### 2.1. High-Fat Diet Induces Obesity and Cardiac Dysfunction in Drosophila

A high-fat diet (HFD) is a major contributor to obesity and cardiovascular disease in humans [15]. To investigate the impact of lipid accumulation on cardiac function in Drosophila, we first examined the effects of HFDs on whole-body lipid metabolism in Drosophila. Obesity is known to increase the risk of cardiovascular disease and impair physical activity [4,16], and in mammals, triglycerides (TGs) are a significant risk factor for obesity and metabolic syndrome [17].

**Figure 1:**
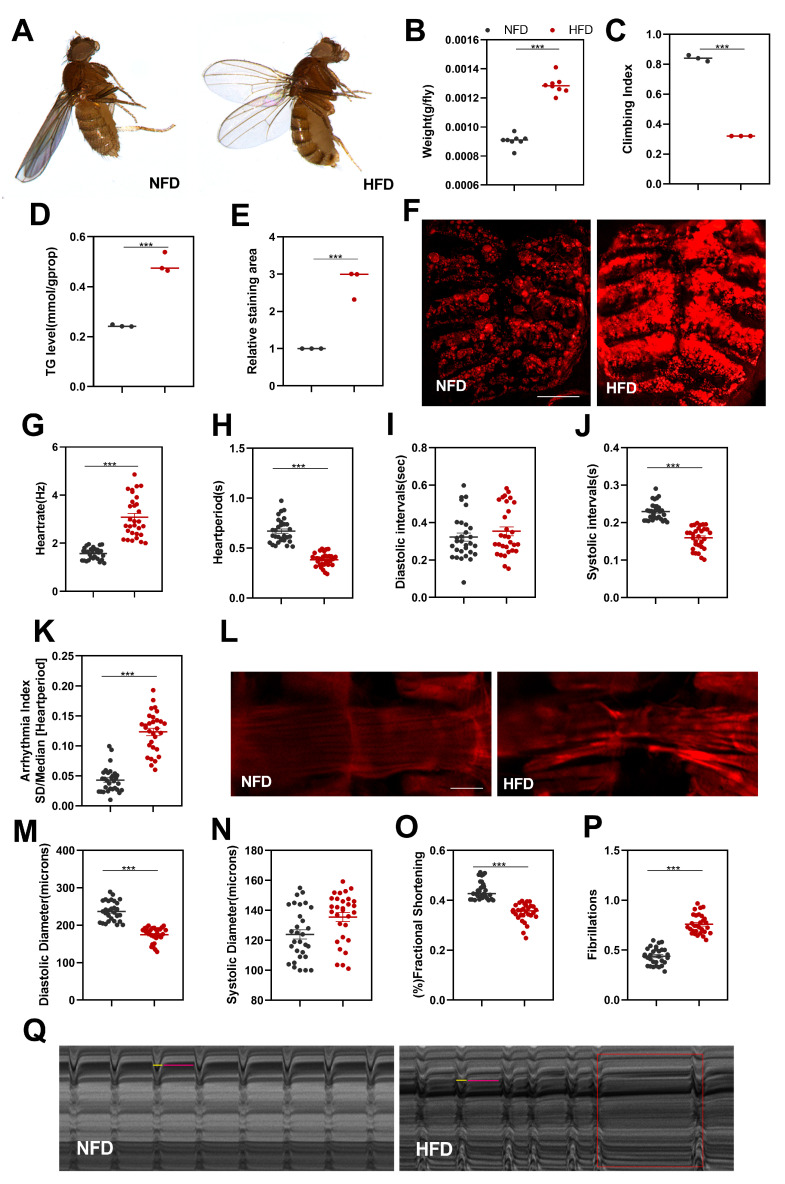
Obesity and cardiac dysfunction in Drosophila after 5 days of HFD in the *W^1118^* group. (**A**) Morphological images of a 10-day-old Drosophila with a protruding abdomen under the same developmental conditions, comparing NFD and HFD groups. (**B**) Body weight measurements of 10-day-old Drosophila. Body weights were determined using an electronic microbalance, with n = 8. Drosophila in the HFD group exhibited significantly higher body weights compared to those in the NFD group. (**C**) Climbing index of Drosophila. The HFD group demonstrated a significantly lower climbing index compared to the NFD group. Climbing index was calculated as the number of Drosophila at the top divided by the total number, with n = 50, and the experiment was repeated three times. (**D**) Whole-body triglyceride (TG) levels in 10-day-old Drosophila. TG levels were markedly elevated in the HFD group compared to in the NFD group, with n = 8, and the experiment was repeated three times. (**E**,**F**) Nile red staining of Drosophila abdomens. Nile red staining revealed increased staining in the HFD group compared to in the NFD group. Quantification of the Nile red staining area was performed, with n = 3. Scale bar = 500 μm (**G**–**O**) Quantification of M-mode parameters in the NFD and HFD groups, including HR, AI, FS, SD, DD, DI, SI, HP, and FL. The sample size for each group was n = 25 ± 5. (**P**) Results of microfilament staining in Drosophila hearts for the HFD and NFD groups. Confocal microscopy was used to visualize ghost pen cyclic peptide-labeled F-actin in Drosophila hearts. Scale bar = 250 μm, with N = 3. (**Q**) Drosophila M-mode diagram. The short yellow line and the long pink line indicate systolic and diastolic intervals, respectively. Red rectangular areas represent arrhythmias. All M-mode recordings were conducted over a 10 s duration. Statistical differences between the two groups were assessed using independent sample *t*-tests. Data are presented as mean, with error bars representing SEM values. Significance levels are denoted as *** *p* < 0.001.

Five days after being placed on an HFD, Drosophila exhibited several notable changes: a significant increase in body weight (Figure 1B), a significant decrease in climbing ability (Figure 1C), a significant rise in TG levels (Figure 1D), and a marked increase in the area of Nile red staining (Figure 1E,F). To evaluate the effects of HFDs on Drosophila cardiac function, we visualized filamentous actin (F-actin) in Drosophila hearts using phalloidin, and M-mode analysis was used to quantify various parameters, including heart rate (HR), cardiac cycle (heart period, HP), diastolic interval (DI), systolic interval (SI), arrhythmia index (AI), diastolic diameter (DD), systolic diameter (SD), fractional shortening (FS), and fibrillation (FL).

The results revealed significant morphological and functional abnormalities in the hearts of Drosophila from the HFD group when compared to those from the NFD group. Specifically, Drosophila cardiac microfilaments in the HFD group exhibited irregularities, breakage, and shortening, whereas those in the normal dietary feeding (NFD) group remained intact and orderly, indicating structural damage to cardiomyocytes in response to an HFD (Figure 1P).

Furthermore, Drosophila in the HFD group displayed significantly higher HR, shorter HP, shorter SI, increased AI, reduced DD, diminished FS, and elevated FL (Figure 1G–O). These findings collectively suggest that an HFD has a profound negative impact on Drosophila cardiac function, characterized by impaired ventricular systolic and diastolic capacities, reduced cardiac output, and cardiac arrhythmias.

### 2.2. HFDs Induce Obesity and Cardiac Dysfunction in Drosophila and Exercise Improves HFD-Induced Obesity and Cardiac Dysfunction

Exercise is a highly effective preventive and therapeutic strategy for addressing both obesity and heart disease [18]. HFDs are known to lead to weight gain and elevated blood-lipid levels, which can negatively impact cardiac function [19]. Exercise exerts its beneficial effects by ameliorating HFD-induced obesity and cardiac dysfunction through multiple mechanisms [20]. Consequently, exercise plays a pivotal role in improving obesity and preventing cardiovascular disease.

**Figure 2:**
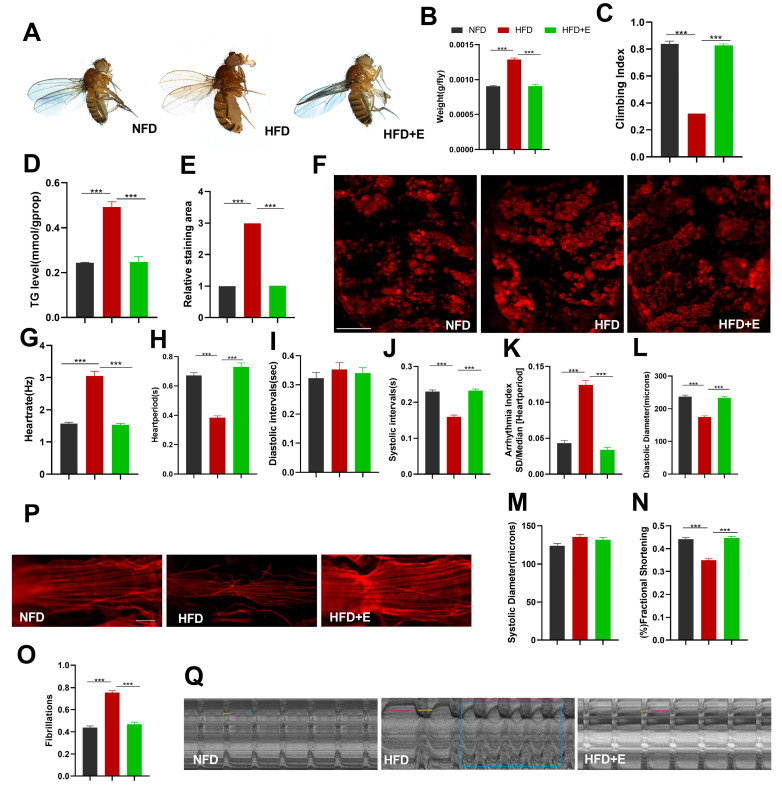
Improvement in obesity and cardiac dysfunction after 5 days of exercise in Drosophila *W^1118^*. (**A**) Morphological images of 10-day-old Drosophila in the NFD, HFD, and HFD+E groups after 5 days of exercise. Drosophila development was identical, but the HFD+E group displayed a smaller protruding abdomen compared to the HFD group. (**B**) Body weight measurements of Drosophila in the NFD, HFD, and HFD+E groups, with n = 8. (**C**) Climbing index of Drosophila in the NFD, HFD, and HFD+E groups. The experiment was repeated three times, with n = 50. (**D**) Whole-body triglyceride (TG) levels of Drosophila, with n = 8. The experiment was repeated three times. (**E**,**F**) Nile red staining of fat in Drosophila abdomens, with n = 3. Scale bar = 500 μm (**G**–**O**) Cardiac M-mode quantification of various parameters, including HR, AI, FS, SD, DD, DI, SI, HP, and FL in the NFD, HFD, and HFD+E groups. The sample size for each group was N = 25 ± 5. (**P**) Results of microfilament staining of Drosophila hearts in the NFD, HFD, and HFD+E groups. Confocal microscopy was used to visualize ghost pen cyclic peptide-labeled F-actin from Drosophila hearts. Scale bar = 250 μm, with n = 3. (**Q**) Drosophila M-mode diagram. Short yellow lines and long pink lines represent intersystolic and diastolic intervals, respectively. The blue rectangular area represents fibrillation. All M-mode recordings were conducted over a 10 s duration. Statistical differences between the three groups were assessed using one-way ANOVA. Data are expressed as mean, with error bars representing SEM values. Significance levels are indicated as *** *p* < 0.001.

The results of this study demonstrated that, in comparison to the HFD group, Drosophila in the HFD+E group exhibited a significantly higher climbing index (Figure 2C), significantly lower triglyceride (TG) levels (Figure 2D), and significantly smaller areas of Nile red staining (Figure 2E,F). These findings strongly indicate that exercise effectively mitigates high-fat-diet-induced obesity and enhances exercise capacity.

To evaluate the impact of exercise on high-fat-diet-induced cardiac dysfunction in Drosophila, M-mode analysis was used to quantify various parameters, including HR, HP, DI, SI, AI, DD, SD, and FS. The results revealed that Drosophila in the HFD+E group exhibited a significant reduction in HR, an increase in HP, an increase in SI, a decrease in AI, an increase in DD, and an increase in FS. These differences were all statistically significant when compared to the effects recorded in the HFD group (Figure 2G–O). These results strongly suggest that exercise effectively ameliorates high-fat-diet-induced cardiac dysfunction in Drosophila.

### 2.3. dfoxo and dsrebp Signaling Factors Play Vital Roles in Exercise-Mediated Amelioration of HFD-Induced Lipid Metabolism

The *foxo* and *srebp* signaling factors are pivotal transcription factors responsible for regulating the synthesis and breakdown of fatty acids [21,22]. HFDs disrupt the balance of *foxo* and *srebp*, resulting in disturbances in fat metabolism [14,23].

**Figure 3:**
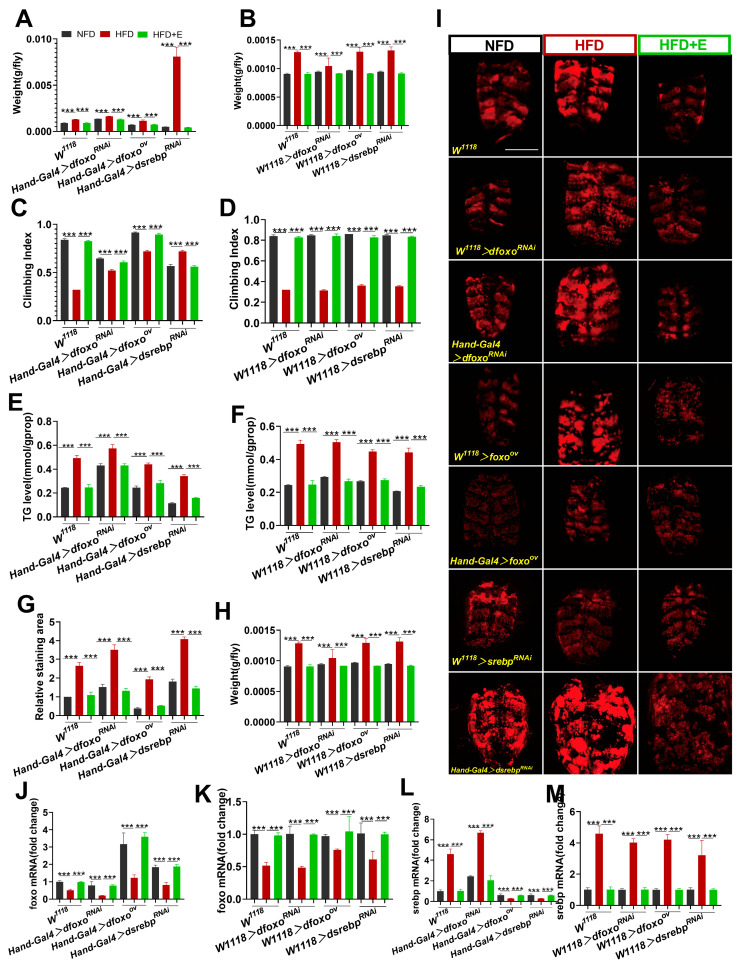
Roles of the *dfoxo* and *dsrebp* signaling factors in the improvement of HFD-induced lipid metabolism with exercise. (**A**) Comparison of body weights in the Hand-Gal4 > *dfoxo*^RNAi^, Hand-Gal4 > dfo*xo*^ov^, and Hand-Gal4 > *dsrebp*^RNAi^ groups under NFD, HFD, and HFD+E conditions. n = 8. (**B**) Comparison of body weights in the W1118 > *dfoxo*^RNAi^, *W^1118^* > *dfoxo*^ov^, and *W^1118^* > *dsrebp*^RNAi^ groups under NFD, HFD, and HFD+E conditions. n = 8. (**C**) Comparison of climbing indices in the Hand-Gal4 > *dfoxo*^RNAi^, Hand-Gal4 > *dfoxo*^ov^, and Hand-Gal4 > *dsrebp*^RNAi^ groups under NFD, HFD, and HFD+E conditions. n = 50; repeated three times. (**D**) Comparison of climbing indices in the W1118 > *dfoxo*^RNAi^, W1118 > *dfoxo*^ov^, and *W^1118^* > *dsrebp*^RNAi^ groups under NFD, HFD, and HFD+E conditions. n = 50; repeated three times. (**E**) Whole-body TG levels in the Hand-Gal4 > *dfoxo*^RNAi^, Hand-Gal4 > *dfoxo*^ov^, and Hand-Gal4 > *dsrebp*^RNAi^ groups under NFD, HFD, and HFD+E conditions. n = 8; repeated three times. (**F**) Whole-body TG levels in the W1118 > *dfoxo*RNAi, W1118 > *dfoxo*ov, and W1118 > *dsrebp*^RNAi^ groups under NFD, HFD, and HFD+E conditions. n = 8; repeated three times. (**G**–**I**) Comparison of Nile red stained areas in the Hand-Gal4 > *dfoxo*^RNAi^, Hand-Gal4 > *dfoxo*^ov^, and Hand-Gal4 > *dsrebp*^RNAi^ groups under NFD, HFD, and HFD+E conditions. n = 3. Scale bar = 500 μm (**J**) Relative expression levels of cardiac *dfoxo* mRNAs in the Hand-Gal4 > *dfoxo*^RNAi^, Hand-Gal4 > *dfoxo*^ov^, and Hand-Gal4 > *dsrebp*^RNAi^ groups under NFD, HFD, and HFD+E conditions. n = 30. (**K**) Cardiac *dfoxo* mRNA relative expression levels in the W1118 > *dfoxo*^RNAi^, W1118 > *dfoxo*ov, and W1118 > *dsrebp*^RNAi^ groups under NFD, HFD, and HFD+E conditions. n = 30. (**L**) Heart *dsrebp* mRNA relative expression levels in the Hand-Gal4 > *dfoxo*^RNAi^, Hand-Gal4 > *dfoxo*^ov^, and Hand-Gal4 > *dsrebp*^RNAi^ groups under NFD, HFD, and HFD+E conditions. n = 30. (**M**) Relative expression levels of *dsrebp* mRNA in the hearts of the W1118 > *dfoxo*^RNAi^, W1118 > *dfoxo*^ov^, and *W^1118^* > *dsrebp*^RNAi^ groups under NFD, HFD, and HFD+E conditions. n = 30. One-way ANOVA was used to test for differences among the three groups; data are expressed as mean, and the SEM values are represented as error lines. *** *p* < 0.001.

The results of this study indicated that body weight, climbing index, triglyceride (TG) levels, and Nile red staining areas were all elevated in the UAS-*dfoxo*^RNAi^, UAS-*dfoxo*ov, and UAS-*dsrebp*^RNAi^ HFD groups, concomitant with a reduction in *dfoxo* mRNA content in all these groups (Figure 3A–K). These findings highlight the potential of HFDs to induce aberrations in lipid metabolism in Drosophila. Moreover, *dsrebp* mRNA content increased in the UAS-*dfoxo*^RNAi^ HFD group, while it decreased in both the UAS-*dfoxo*^ov^ and UAS-*dsrebp*^RNAi^ groups (Figure 3J–M). These results underscore the roles of the *dfoxo* and *dsrebp* genes in HFD-induced obesity and cardiac dysfunction in Drosophila. Specifically, the *dfoxo* gene exerts an inhibitory effect on the expression of the dsrebp gene, while the *dsrebp* gene promotes the synthesis and accumulation of lipids in Drosophila cardiomyocytes.

Following an HFD, in comparison to the NFD group, the UAS-*dfoxo*^ov^ and UAS-*dsrebp*^RNAi^ groups displayed reduced body weight, TG levels, and Nile red stained area, along with elevated climbing indices and *dfoxo* mRNA content. Conversely, the UAS-*dfoxo*^RNAi^ group exhibited increased body weight, TG levels, and Nile red stained area, alongside reduced climbing indices and *dfoxo* mRNA content (Figure 3A–K). These findings indicate that the overexpression or knockdown of the *dfoxo* or *dsrebp* genes can modulate Drosophila’s response to an HFD. The overexpression of *dfoxo* genes or knockdown of the *dsrebp* gene can mitigate the detrimental effects of HFDs on Drosophila, while the knockdown of *dfoxo* genes can exacerbate these effects.

Comparing the HFD group to the HFD+E group, Drosophila in the HFD+E group exhibited significantly lower body weight, a significantly higher climbing index, significantly lower TG levels, a smaller relative Nile red staining area, significantly elevated *dfoxo* mRNA expression, and significantly reduced *dsrebp* mRNA expression (Figure 3A–M). These results highlight that exercise can effectively ameliorate HFD-induced obesity and lipid metabolism in Drosophila, concurrently restoring *dfoxo* gene levels and suppressing *dsrebp* gene expression. Consequently, it is inferred that *dfoxo* and *dsrebp* signaling factors play crucial roles in improving lipid metabolism in Drosophila subjected to HFDs through exercise.

### 2.4. dfoxo and dsrebp Signaling Factors Play Important Roles in the Improvement of HFD-Induced Cardiac Function with Exercise

HFDs lead to cardiac dysfunction and increase the risks of cardiovascular disease [24]. To explore the mechanisms of the *dfoxo* and *dsrebp* signaling factors in the improvement of high-fat-induced cardiac function with exercise, we examined Drosophila cardiac function.

**Figure 4:**
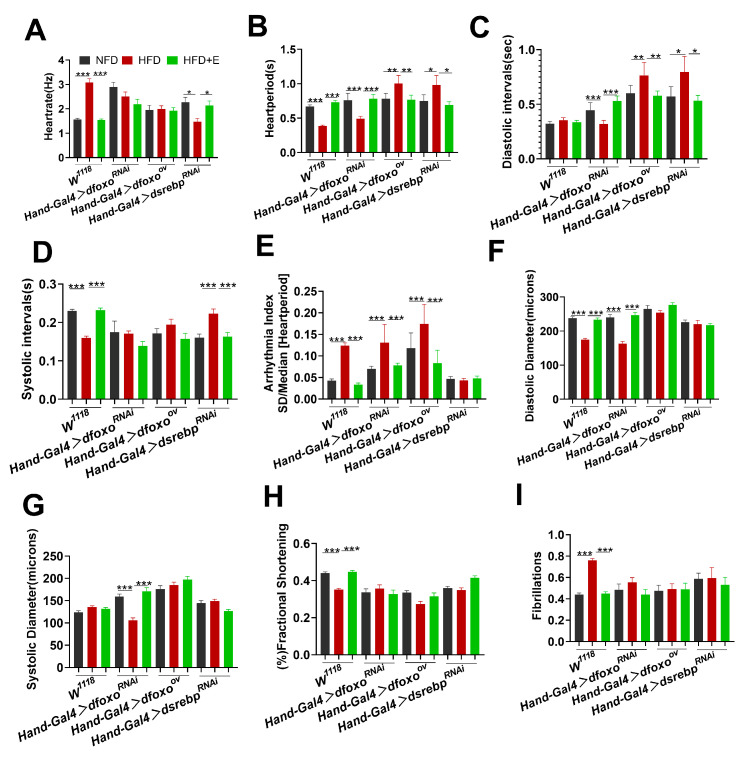
Roles of the *dfoxo* and *dsrebp* signaling factors in the improvement of high-fat-induced cardiac function with exercise. (**A**–**I**) M-mode quantification of cardiac function in the hearts of Drosophila from the NFD, HFD, and HFD+E groups with manipulated *dfoxo* and *dsrebp* expression. Parameters measured include HR, AI, FS, SD, DD, DI, S, HP, and FL. Sample size n = 25 ± 5. One-way ANOVA was used to assess differences between the three groups. Data are presented as mean, with error bars indicating SEM values. * *p* < 0.05, ** *p* < 0.01, *** *p* < 0.001. UAS-dfoxoRNAi, UAS-*dfoxo*^ov^, and UAS-*dsrebp*^RNAi^ lines were crossed with Drosophila W1118 to establish the respective transgenic Drosophila strains.

In comparison to the NFD group, Drosophila in the HFD group exhibited several notable changes in cardiac parameters. Specifically, the HFD group showed reductions in HR, HP, and an increase in DI and SI. These findings suggest that the HFD had a detrimental impact on cardiac function. Interestingly, when we knocked down the expression of *dsrebp* in the hearts of Drosophila in the HFD group (Figure 4A–D), we found that the results showed exactly the opposite state, indicating that the knockdown of the *dsrebp* gene contributed to mitigating HFD-induced cardiac dysfunction. Conversely, when we knocked down *dfoxo* in the hearts of Drosophila in the HFD group, we observed a further deterioration in cardiac function. This was evident through decreases in HP, DI, DD, and SD, coupled with an increase in AI (Figure 4A–I). These results imply that knocking down *dfoxo* exacerbates the abnormal cardiac function in Drosophila exposed to an HFD. In contrast, upon overexpressing *dfoxo* in the hearts of Drosophila in the HFD group (Figure 4B–E), we observed improvements in cardiac parameters. This was reflected in elevated HP, DI, and AI, suggesting that *dfoxo* overexpression alleviates the abnormal cardiac state in Drosophila hearts under the influence of an HFD. These findings highlight a protective role for *dfoxo* in Drosophila hearts exposed to an HFD. Furthermore, exercise was found to enhance both the systolic and diastolic capacities of Drosophila hearts, as well as stabilize heart rhythm. These effects were attributed to the regulation of the expressions and activities of the *dfoxo* and *dsrebp* signaling factors. Based on these results, we propose that the *dfoxo* and *dsrebp* signaling factors play pivotal roles in ameliorating HFD-induced cardiac dysfunction in Drosophila through exercise.

**Figure 5:**
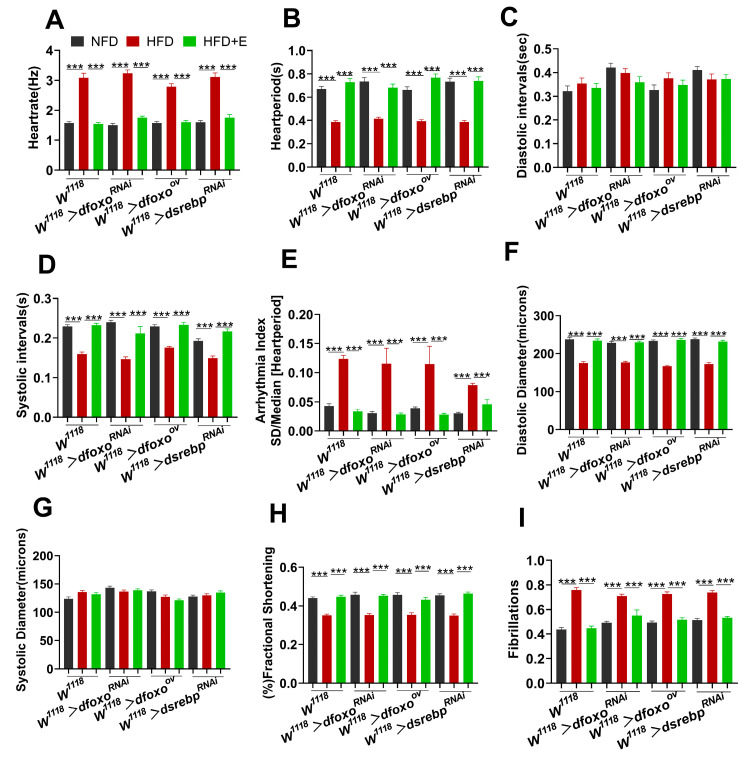
Hybridization of UAS-*dfoxo*^RNAi^, UAS-*dfoxo*^ov^, and UAS-*dsrebp*^RNAi^ with Drosophila *W^1118^*. (**A**–**I**) Quantitative analyses of HR, AI, FS, SD, DD, DI, SI, HP, and FL in response to NFD, HFD, and HFD+E treatments using Drosophila heart M-mode in the *W1118* > *dfoxo*^RNAi^, *W^1118^* > *dfoxo*^ov^, and *W^1118^* > *dsrebp*^RNAi^ experimental groups. n = 25 ± 5. Data are expressed as mean, with error bars representing SEM values. Significance levels are indicated as *** *p* < 0.001.

In order to eliminate potential genetic background interference, we conducted homozygous backcrossing of all transgenic Drosophila strains with Drosophila W1118 for a minimum of six generations. Subsequently, we conducted cardiac function measurements, and no significant differences in cardiac function were observed among the *W^1118^* > *dfoxo*^RNAi^, *W^1118^* > *dfoxo*^ov^, or *W^1118^* > *dsrebp*^RNAi^ groups (Figure 5A–I). These results indicate that genetic background interference had been effectively mitigated.

The following statement explains the process of generating transgenic Drosophila strains by crossing them with the *W^1118^* strain. To ensure the reliability of the experimental results and eliminate potential genetic background effects, rigorous measures were taken, including multiple generations of homozygous backcrossing. The outcome of this process confirmed that the observed effects were not influenced by genetic-background variations.

**Figure 6:**
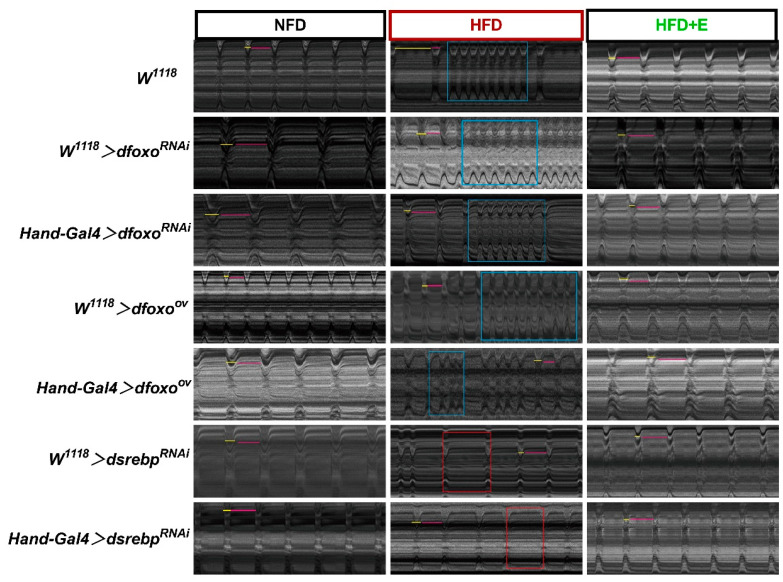
Drosophila M-mode diagrams. M-mode plots depict the cardiac activity of Drosophila in various experimental groups, NFD, HFD, and HFD+E, under the influence of Hand-Gal4 > *dfoxo*^RNAi^, Hand-Gal4 > *dfoxo*^ov^, and Hand-Gal4 > *dsrebp*^RNAi^. In these diagrams, short yellow lines denote the intersystolic intervals, while long pink lines represent diastolic intervals. The red rectangular areas highlight instances of arrhythmias, and the blue rectangular areas indicate occurrences of fibrillation. All M-mode figure time intervals are standardized to 10 s.

We observed a noticeable level of disorganization and abnormality in the M-mode plots of Drosophila following an HFD regimen, irrespective of whether there was a knockdown or overexpression of the *foxo* gene, or a knockdown of the *srebp* gene (Figure 6). It is conceivable that an M-mode plot may primarily provide a macroscopic overview, while more pronounced cardiac damage might become evident in more detailed data presentations.

Staining the microfilaments of Drosophila hearts with the ghost pen cyclic peptide allowed us to visualize the morphology and arrangement of cardiomyocytes. Our results revealed that microfilament staining of Drosophila cardiomyocytes, following knockdown of the *foxo* gene, exhibited significant disorganization and breakage under the conditions of HFD feeding. This indicates that the suppression of *foxo* expression exacerbates the cardiomyocyte damage induced by a high-fat diet in Drosophila, resulting in reduced stability and integrity of these cells.

**Figure 7:**
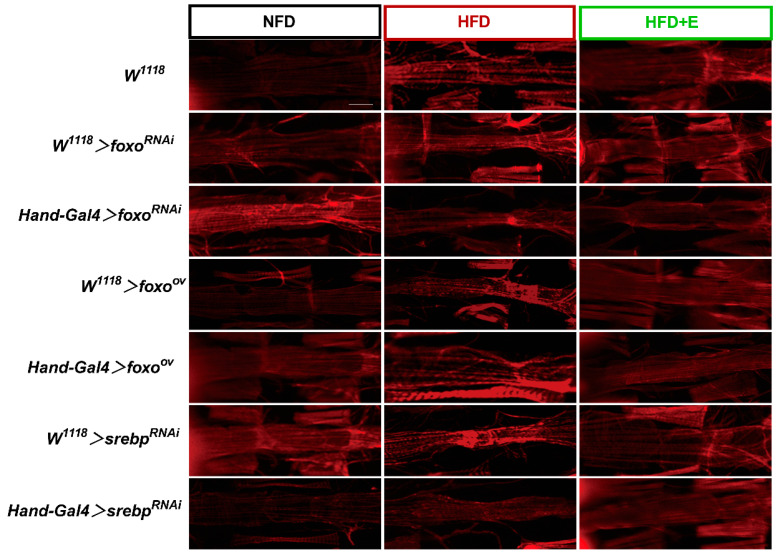
Staining of Drosophila heart with ghost pen cyclic peptide. Results of microfilament staining of NFD, HFD, and HFD+E Drosophila hearts in the Hand-Gal4 > *dfoxo*^RNAi^, Hand-Gal4 > *dfoxo*^ov^, and Hand-Gal4 > *dsrebp*RN^Ai^ groups. n = 3. Scale bar = 250 μm.

Conversely, when we overexpressed the *foxo* gene and knocked down the *srebp* gene, microfilament staining of Drosophila cardiomyocytes exhibited remarkable orderliness and continuity. This suggests that the overexpression of *foxo* and the suppression of *srebp* expression can ameliorate HFD-induced injuries in Drosophila cardiomyocytes, ultimately enhancing their stability and integrity (Figure 7).

## 3. Discussion

Cardiovascular diseases (CVDs) represent a significant global health challenge, as they are responsible for approximately 18 million annual deaths [25]. The emergence and progression of these diseases are multifactorial, with diet representing a crucial, controllable factor [26]. Dietary choices exert a profound influence, not only on metabolic, endocrine, and immune functions, but also on the structural and functional aspects of the heart, thereby directly impacting cardiovascular health [27].

In recent years, mounting evidence has highlighted the detrimental effects of a high-fat diet (HFD), a prevalent unhealthy dietary pattern, on the risk of cardiovascular disease [28]. HFDs have been linked to a cascade of metabolic abnormalities, including obesity, dyslipidemia, inflammation, and oxidative stress, which, in turn, trigger various cardiac dysfunctions, such as cardiomyocyte damage and arrhythmia [29]. Nevertheless, the intricate molecular mechanisms through which an HFD impacts cardiac function remain elusive. Specifically, the mechanisms through which an HFD affects two pivotal transcription factors, *dfoxo* and *dsrebp*, and their roles in regulating lipid metabolism and cardiac function in Drosophila, remain subjects of inquiry. Addressing these questions holds significant importance for unraveling the intricate relationship between high-fat diets and cardiovascular diseases, as well as for identifying novel targets in the quest for preventing and treating these conditions.

In our study, we delved into the impacts of regular exercise on HFD-induced obesity and cardiac dysfunction in Drosophila, with a particular focus on the involvement of the *dfoxo* and *dsrebp* signaling factors. Our findings revealed that Drosophila, when subjected to an HFD regimen, exhibited the development of obesity along with cardiac dysfunction.

Specifically, the HFD led to increases in both body weight and triglyceride (TG) levels in Drosophila. This phenomenon can be attributed to surplus energy intake, which, when exceeding expenditure, is converted into TGs and subsequently stored, resulting in elevations in both body weight and TG levels [30]. HFDs, rich in fat and grease, contribute to increased energy intake in Drosophila, thus promoting the synthesis and accumulation of TGs [31].

However, the good news is that the adverse effects of obesity and cardiac dysfunction induced by HFDs in Drosophila were mitigated following an exercise intervention. This implies that exercise holds promise in alleviating the impairment of cardiac function caused by HFDs. More precisely, exercise played a role in reducing body weight and TG levels in Drosophila. This can be explained by the fact that exercise increases the energy expenditure in Drosophila, consequently curbing the storage and accumulation of TGs [32].

Furthermore, it is important to note that HFDs have been reported to detrimentally affect the functionality and structure of Drosophila cardiomyocytes [33], while exercise has been shown to ameliorate such HFD-induced cardiac function impairments [34].

Our investigation showed that lipid-metabolism disorders and cardiac dysfunction in Drosophila subjected to HFD feeding were partially ameliorated through the modulation of both *dfoxo* and *dsrebp* gene expressions within Drosophila hearts. These findings underscore the significance of the *dfoxo* and *dsrebp* genes as pivotal molecular factors contributing to HFD-induced obesity and cardiac dysfunction.

Upon HFD feeding, we observed a decrease in *dfoxo* mRNA levels and an increase in *dsrebp* mRNA levels in Drosophila. Subsequently, when we downregulated *dfoxo* expression in the hearts of Drosophila within the HFD group, several noteworthy consequences ensued. Lipid metabolism was disrupted in Drosophila under HFD conditions, and the cardiac parameters of HP, DI, DD, and SD were all diminished, accompanied by an elevated AI.

It is worth noting that foxo functions as a transcription factor, regulating a diverse array of genes associated with metabolism and growth [35]. Under normal circumstances, *foxo* acts to inhibit the synthesis of fatty acids and triglycerides, while promoting fatty-acid oxidation and catabolism [36]. However, in Drosophila consuming an HFD, insulin signaling becomes heightened, leading to the phosphorylation of *foxo* [37]. This phosphorylation results in the inactivation of *foxo*’s ability to regulate its target genes, ultimately culminating in an increase in fat synthesis and a decrease in lipolysis in Drosophila [38,39].

Consequently, the knockdown of foxo expression in the hearts of HFD-fed Drosophila led to a weakening of *foxo*’s regulatory effect on its target genes. This, in turn, exacerbated lipid-metabolism disorders and obesity in Drosophila subjected to a high-fat diet. The repercussions of lipid-metabolism disorders and obesity extend beyond the metabolic realm, adversely affecting the Drosophila cardiovascular system and causing structural and functional damage to Drosophila cardiomyocytes.

These findings collectively emphasize the critical roles of the *dfoxo* and *dsrebp* genes, and their intricate interplay, in the context of HFD-induced obesity and cardiac dysfunction in Drosophila.

The notable increase in *dsrebp* mRNA expression observed following HFD consumption signifies that an HFD exacerbates the upregulation of *dsrebp* mRNA in Drosophila, rendering them more susceptible to excessive fat accumulation. Subsequently, when we intervened by knocking down the *dsrebp* gene, we observed an amelioration in the symptoms associated with abnormal lipid metabolism and cardiac function in HFD-fed Drosophila.

*dsrebp* exhibited upregulation in response to high-fat dietary conditions, which, in turn, stimulated lipid synthesis and accumulation within Drosophila cardiomyocytes. The accumulation of these excess lipids significantly disrupted the structure and function of cardiomyocytes, resulting in various abnormalities, such as an increased HR, a shortened HP, a reduced DI, an elevated AI, a diminished DD, a reduced FS, and an increased incidence of FL.

The knockdown of *dsrebp* expression served to inhibit the exacerbated *dsrebp* mRNA gene expression induced by HFDs. Consequently, this reduction in *dsrebp* expression led to a decrease in lipid synthesis and accumulation within Drosophila cardiomyocytes. As a result, the structural and functional aspects of cardiomyocytes improved, alleviating the phenotypic manifestations of cardiac dysfunction.

Collectively, these findings underscore the pivotal roles and significance of the *dfoxo* and *dsrebp* signaling factors in the context of exercise-mediated improvements in HFD-induced obesity and cardiac dysfunction in Drosophila.

Presently, the scientific community faces the challenge of accurately defining the specific mode of exercise in Drosophila (whether it is aerobic or anaerobic). This ambiguity makes it difficult to conclusively attribute the beneficial effects of exercise on Drosophila lipid metabolism to either anaerobic or aerobic exercise.

In summary, our study sheds light on the underlying mechanism through which regular exercise modulates the *dfoxo*/*dsrebp* pathway, thereby ameliorating the adverse impacts of HFD-induced obesity and cardiac dysfunction in Drosophila. This research offers new avenues for further understanding the intricate molecular connections between high-fat diets and cardiovascular disease.

Furthermore, our study provides valuable insights into prevention and intervention strategies for obesity and cardiovascular disease in humans. These strategies encompass the regulation of dietary fat intake, the maintenance of appropriate levels of physical exercise, and the monitoring of the *dfoxo* and *dsrebp* signaling pathways within the heart to sustain normal cardiac function.

**Figure 8:**
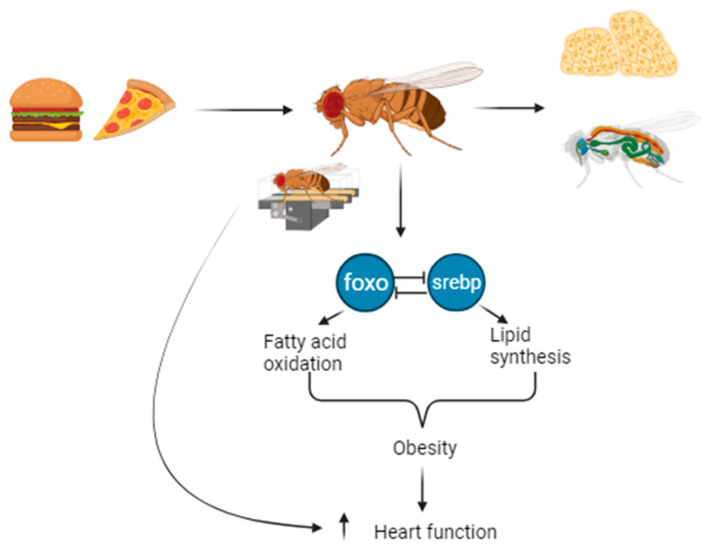
Impacts of regular exercise on obesity and cardiac function in Drosophila through *dfoxo* and *dsrebp* signaling.

Our findings suggest that regular exercise has the potential to mitigate high-fat-diet-induced obesity and cardiac dysfunction in Drosophila by modulating the *dfoxo* and *dsrebp* signaling pathways. This discovery provides novel insights and a rational basis for further comprehension of the pathogenesis of obesity and cardiovascular diseases in humans, as well as for identifying effective preventative and therapeutic strategies. However, the effects of high-fat diets on human obesity and cardiac dysfunction are complex and multifactorial, involving not only metabolic and molecular alterations, but also environmental and behavioral factors [40]. The Centers for Disease Control and Prevention (CDC) states that high-fat diets impair insulin sensitivity and lipid metabolism, and thus increase the risk of cardiovascular disease [41]. Therefore, our findings may provide new ideas for the development of drugs or nutritional supplements targeting signaling pathways such as foxo and srebp to prevent or treat the metabolic disorders and cardiovascular diseases caused by high-fat diets. In addition, this study provides the public with a simple and effective health recommendation to stay fit through regular exercise. Our findings have the potential to influence the understanding of the molecular mechanisms of obesity and cardiovascular disease in humans, as well as the development of prevention and treatment strategies based on exercise interventions. However, we also recognize that there are significant differences between Drosophila and humans in terms of physiology, anatomy, genetics, and environmental factors, which may limit the applicability of our findings to humans [42]. Therefore, further studies are needed to validate our findings in mammalian models or human subjects. In conclusion, this study presents compelling evidence that regular exercise can effectively modulate the *dfoxo*/*dsrebp* pathway, ultimately mitigating the consequences of high-fat-diet-induced obesity and cardiac dysfunction in Drosophila. These findings not only contribute to a better understanding of exercise’s impact on human health, but also provide a theoretical basis upon which to utilize the Drosophila model to investigate the effects of exercise on human well-being (Figure 8).

## 4. Materials and Methods

### 4.1. Drosophila Strains and Rearing

Drosophila strains, including *W^1118^*, Hand-Gal4, UAS-*dfoxo*^ov^, and UAS-*dsrebp*^RNAi^, were procured from the Drosophila Inventory Center, Bloomington, while the UAS-*dfoxo*^RNAi^ strain was obtained from the Drosophila Center at Tsinghua University. To eliminate potential genetic background interference, UAS-*dfoxo*^RNAi^, UAS-*dfoxo*^ov^, and UAS-*dsrebp*^RNAi^ were crossed with the Drosophila *W^1118^* strain.

Additionally, UAS-*dfoxo*^RNAi^ was crossed with Drosophila Hand-Gal4 to generate Drosophila with cardiac-specific *dfoxo* knockdown. UAS-*dfoxo*^ov^ was crossed with Drosophila Hand-Gal4 to create Drosophila with cardiac-specific *dfoxo* overexpression, and UAS-*dsrebp*^RNAi^ was crossed with Hand-Gal4 Drosophila to induce cardiac-specific *dsrebp* knockdown.

The high-fat diet was designated as “HFD”, the normal diet was referred to as “NFD”, and the combination of a high-fat diet and exercise training was termed “HFD+E”.

Drosophila were maintained in a climate-controlled environment at a constant temperature and humidity (25 °C and 50% humidity) with a 12 h light/12 h dark photoperiod. Unless otherwise specified, female virgin flies were utilized in the experiments. The high-fat medium was formulated by combining 30% coconut oil with standard medium, and the obese Drosophila phenotype was induced by subjecting the Drosophila to a high-fat diet for 5 days [34,43].

### 4.2. Body Weight and Triglyceride Measurement

Drosophila were weighed using an electronic microbalance (Uni Group, AUW220D, shimadzu, Japan), and the weight of each individual Drosophila was recorded. Triglyceride (TG) concentrations were determined using an insect TG ELISA kit (mlbio, Nanjing, China) in accordance with the manufacturer’s instructions [44].

### 4.3. Negative Geotaxis Assay

Climbing experiments were conducted following established protocols [45]. The climbing apparatus consisted of an 18 cm glass tube with an inner diameter of 2.8 cm. To prevent fruit flies from escaping, while still allowing for proper gas exchange, a sponge was placed at the end of the tube. Due to the innate negative geotaxis behavior of fruit flies, when they were gently tapped to the bottom of the tube, they would instinctively climb upward toward the top. Fruit flies were allowed to climb for 8 s after being tapped down, and the height reached by each fruit fly was measured. Throughout the experiment, a video camera recorded the climbing behavior; the 4th, 5th, and 6th climbing images at the end of 8 s were intercepted; and the number of flies reaching the top was counted. The climbing index was calculated as the number of Drosophila in the uppermost region divided by the total number of flies [34].

### 4.4. Phalloidin

Preparation of semi-intact Drosophila hearts: semi-intact Drosophila hearts were prepared following the previously established protocol [46]. The anterior dorsal vessel was swiftly immersed in a relaxation buffer containing 10 mM EGTA to replace the artificial hemolymph (ADH). Subsequently, the samples were fixed with 4% paraformaldehyde for a duration of 25 min. Washing steps: the fixation buffer (4% paraformaldehyde) was discarded, and the samples underwent ten washes, each lasting 30 min, with phosphate-buffered saline (PBS). Staining: to facilitate staining, a drop of ghost pen cyclic peptide dye was applied to the sample, which was then incubated on a shaker and shielded from light for 10 min. Final wash: after staining, the dye was removed, and the samples were subjected to additional PBS washes as needed. Sample mounting: slides were prepared, and the treated samples were carefully transferred onto these slides. Coverslips were applied to cover the samples securely. Image acquisition: finally, images were captured using a Leica stereomicroscope (Leica; Wetzlar; Germany).

### 4.5. Quantification of Nile Red Staining

The head and tail of Drosophila were removed in PBS, and both the intestines and ovaries were carefully dissected from the Drosophila and fixed in 4% paraformaldehyde for 20 min. After fixation, they were washed three times with PBS. Staining was carried out using a fresh Nile red dye solution (1 mL PBS and 1 μL Nile red solution) for 20 min, followed by rinsing with PBS. It is important to note that the Drosophila viscera should be gently removed to prevent fat entrapment and minimize staining artifacts [47]. Subsequently, samples were examined and imaged under a fluorescence microscope, and the lipid area was quantified using ImageJ software (Version number: 1.53).

### 4.6. M-Mode Cardiac Function Assay

Drosophila hearts were partially exposed, and their heartbeats were recorded using an EM-CCD high-speed camera (30-s video, >120 frame rate), with data captured and analyzed using HC Image software (Version number: 4.2.1.0). The assessment of Drosophila cardiac function followed the methodology outlined in a previous study by Martin Fink [48]. Quantitative measurements of heart rate (HR), cardiac cycle (heart period, HP), diastolic interval (DI), systolic interval (SI), arrhythmia index (AI), diastolic diameter (DD), systolic diameter (SD), fractional shortening (FS), and fibrillations (FL) were performed using a semi-automated optical heartbeat analysis approach. Each sample group consisted of approximately 25 flies.

### 4.7. Athletic Training Equipment and Programs

Consistent with the previously established program [49], all Drosophila were fed a normal diet (NFD) for 5 days. Drosophila in the high-fat diet (HFD) group were fed a high-fat diet for 5 days starting on day 6, while Drosophila in the HFD+E group underwent training for 5 days starting on day 6, with all Drosophila concluding their interventions on day 10. The exercise regimen involved subjecting Drosophila to 2 h of daily exercise for 5 days. During the exercise training intervention, Drosophila were placed in glass tubes without access to food for 2 h. Drosophila in the NFD and HFD groups were also placed in glass tubes without food for 2 h to ensure that all Drosophila were exposed to the same environmental conditions.

### 4.8. Real-Time Quantitative PCR

Thirty Drosophila heart samples were collected from each group. Total RNA was extracted from the lysate after homogenization using Trizol (Invitrogen, CA, USA) reagent, following the manufacturer’s protocol. The primers used for gene expression analysis were as follows: foxo (F: TCTTCGTAGCAGTCACGTTGT; R: GTTGTTGTTTTTTGAGCGAAATCCA), srebp (F: GCAGTTCCTTCGTTTTCTTTTC; R: GGCTTCCATTTCCAGTCAGTT), and rp49 (F: CTAAGCTGTCGCACAAATGG; R: AACTTCTTGAATCCGGTGGGG).

### 4.9. Statistical Analysis

Figures were generated using GraphPad Prism 8 software. Statistical analyses were conducted using the Statistical Package for the Social Sciences (SPSS) for Windows version 21.0 (SPSS Inc., Chicago, IL, USA). All data are presented as mean ± SEM. One-way ANOVA was employed to identify differences between the Drosophila NFD, HFD, and HFD+E groups with the same genetic backgrounds. Independent sample *t*-tests were used to identify differences between two groups. Data are expressed as mean, with error bars representing SEM values, and with significance levels denoted as * *p* < 0.05, ** *p* < 0.01, *** *p* < 0.001.

## Data Availability

Not applicable.
